# Characterisation of the Antibiotic Profile of *Lysobacter capsici* AZ78, an Effective Biological Control Agent of Plant Pathogenic Microorganisms

**DOI:** 10.3390/microorganisms9061320

**Published:** 2021-06-17

**Authors:** Francesca Brescia, Anthi Vlassi, Ana Bejarano, Bernard Seidl, Martina Marchetti-Deschmann, Rainer Schuhmacher, Gerardo Puopolo

**Affiliations:** 1 Research and Innovation Centre, Department of Sustainable Agro-Ecosystems and Bioresources, Fondazione Edmund Mach, 38098 San Michele all’Adige, Italy; francesca.brescia@unito.it (F.B.); ana.bejaranoramos@unitn.it (A.B.); 2 Department of Agricultural, Forest and Food Sciences (DISAFA), University of Torino, Largo Paolo Braccini 2, 10095 Grugliasco, Torino, Italy; 3 Department of Agrobiotechnology (IFA-Tulln), Institute of Bioanalytics and Agro-Metabolomics, University of Natural Resources and Life Sciences Vienna (BOKU), 3430 Tulln, Austria; anthi.vlassi@boku.ac.at (A.V.); bernhard.seidl@boku.ac.at (B.S.); rainer.schuhmacher@boku.ac.at (R.S.); 4 Center of Agriculture, Food, Environment, University of Trento, 38098 San Michele all’Adige, Italy; 5 Institute of Chemical Technologies and Analytics, TU Wien (Vienna University of Technology), 1060 Vienna, Austria; martina.marchetti-deschmann@tuwien.ac.at

**Keywords:** *Lysobacter capsici*, WAP-8294A2, dihydromaltophilin (HSAF), 2,5-diketopiperazines, MALDI-qTOF-MSI, UHPLC-HRMS/MS, biological control, biopesticides

## Abstract

Determining the mode of action of microbial biocontrol agents plays a key role in their development and registration as commercial biopesticides. The biocontrol rhizobacterium *Lysobacter capsici* AZ78 (AZ78) is able to inhibit a vast array of plant pathogenic oomycetes and Gram-positive bacteria due to the release of antimicrobial secondary metabolites. A combination of MALDI-qTOF-MSI and UHPLC-HRMS/M was applied to finely dissect the AZ78 metabolome and identify the main secondary metabolites involved in the inhibition of plant pathogenic microorganisms. Under nutritionally limited conditions, MALDI-qTOF-MSI revealed that AZ78 is able to release a relevant number of antimicrobial secondary metabolites belonging to the families of 2,5-diketopiperazines, cyclic lipodepsipeptides, macrolactones and macrolides. In vitro tests confirmed the presence of secondary metabolites toxic against *Pythium ultimum* and *Rhodococcus fascians* in AZ78 cell-free extracts. Subsequently, UHPLC-HRMS/MS was used to confirm the results achieved with MALDI-qTOF-MSI and investigate for further putative antimicrobial secondary metabolites known to be produced by *Lysobacter* spp. This technique confirmed the presence of several 2,5-diketopiperazines in AZ78 cell-free extracts and provided the first evidence of the production of the cyclic depsipeptide WAP-8294A2 in a member of *L. capsici* species. Moreover, UHPLC-HRMS/MS confirmed the presence of dihydromaltophilin/Heat Stable Antifungal Factor (HSAF) in AZ78 cell-free extracts. Due to the production of HSAF by AZ78, cell-free supernatants were effective in controlling *Plasmopara viticola* on grapevine leaf disks after exposure to high temperatures. Overall, our work determined the main secondary metabolites involved in the biocontrol activity of AZ78 against plant pathogenic oomycetes and Gram-positive bacteria. These results might be useful for the future development of this bacterial strain as the active ingredient of a microbial biopesticide that might contribute to a reduction in the chemical input in agriculture.

## 1. Introduction

Plant diseases caused by pathogenic bacteria, fungi and oomycetes are a major factor for crop losses and account for billions of dollars in annual economic losses [1]. These plant pathogenic microorganisms are commonly controlled using synthetic chemical pesticides. However, the large-scale and frequent use of chemicals in agriculture led the EU to introduce regulations that promoted a more sustainable use of synthetic chemical pesticides [2]. Consequently, the need for environmentally friendly pesticides has emerged. Alternative approaches to chemical pesticides include the application of microbial biocontrol agents (BCAs) as biopesticides [3]. However, BCAs need to be registered as plant protection products to be applied in crop production [4,5,6]. In this context, deciphering the modes of action of BCAs and identifying their antimicrobial metabolites are two important aspects that might be unravelled through functional studies [3].

Among plant beneficial rhizobacteria, members of the genus *Lysobacter* have been evaluated as BCAs in greenhouse and field trials against plant diseases caused by bacteria, fungi and oomycetes [7,8,9]. The inhibitory activity of *Lysobacter* spp. relies on the production of both lytic enzymes and a plethora of secondary metabolites with antibacterial and antifungal activity [10]. Regarding secondary metabolites with antimicrobial activity, *L. antibioticus* strains are able to produce phenazine antibiotic compounds with a strong antibacterial and antifungal activity [11]. *Lysobacter* spp. are also characterised by a vast array of antimicrobial secondary metabolites with more complex chemical structures. For instance, the cyclopeptides lysocin E, lysobactin, the WAP-8294A compound family as well as tripropeptins,, are some antibacterial peptides produced by *Lysobacter* spp. [12]. Moreover, *L. enzymogenes* strains are able to produce antimicrobial compounds belonging to the family of the polycyclic tetramic acid macrolactams [13,14,15,16]. Among these, dihydromaltophilin or heat stable antifungal factor (HSAF), isolated from *L. enzymogenes* C3, showed great potential in controlling plant pathogens [17]. Other polycyclic tetramic acid macrolactams, such as alteramide B, xanthobaccins and catacandins, showed specific antifungal activity and little or no antibacterial activity [13,14,16].

*Lysobacter capsici* AZ78 (AZ78), isolated from the rhizosphere of tobacco plants, has been studied for its ability to effectively control *Phytophthora infestans* and *Plasmopara viticola* on tomato and grapevine plants, respectively [18,19,20]. Its efficacy in controlling these plant pathogenic oomycetes and its ability to resist copper made AZ78 an interesting candidate for the development of a biopesticide [21]. As a consequence, particular attention has been given to determine its mode of action. A dual RNA-Seq approach indicated that the AZ78 mode of action might be related to its ability to establish a necrotrophic interaction of bacterial mycophagy [22]. During this interaction, AZ78 upregulates several genes involved in the biosynthesis of secondary metabolites with antibiotic activities.

Indeed, AZ78 showed in vitro inhibitory activity against several important plant pathogenic fungi and oomycetes (e.g., *Botrytis cinerea*, *Pythium ultimum*) and several Gram-positive plant pathogenic bacteria (e.g., *Rhodococcus fascians)* [20]. When grown in nutrient-rich conditions, AZ78 produced an array of 2,5-diketopiperazines (e.g., cyclo(L-Pro-L-Tyr)), some of which showed inhibitory activity against plant pathogenic microorganisms [19,23]. Recently, the Matrix-Assisted Laser Desorption/Ionisation-Orthogonal Time of Flight Mass Spectrometric Imaging (MALDI-qTOF-MSI) technique allowed the detection of signals pointing to the release of the antimicrobial HSAF, maltophilin and WAP-8294A2 by AZ78 [24]. Besides these non-volatile metabolites, the arsenal of AZ78 is also rich in volatile compounds toxic against plant pathogenic microorganisms, such as pyrazines and NH_3_, that showed antifungal and antioomycete activity [25,26].

Given the importance that secondary metabolites might have for the registration of a BCA, we aimed to accurately identify and characterise the secondary metabolites with inhibitory activity produced by AZ78. To do this, we carried out functional experiments where MALDI-qTOF-MSI and ultra-high pressure liquid chromatography–high resolution tandem mass spectrometry (UHPLC-HRMS/MS) were combined with in vitro tests against plant pathogenic microorganisms (*P.* *ultimum, Pl. viticola* and *R. fascians*).

## 2. Materials and Methods 

### 2.1. Maintenance of Microorganisms

Bacterial strains were stored at length in glycerol 40% at −80 °C and routinely grown on Luria Bertani Agar (LBA) composed of LB (25 g/L *w/v*; Sigma-Aldrich, St. Louis, MO, USA) and Agar Technological n °1 (16 g/L *w/v*; Oxoid, Basingstoke, UK) in Petri dishes (90 mm diameter). LBA diluted 1:10 (LBA 1:10), consisting of LB (2.5 g/L, *w/v*) and Agar Technological n °1 (16 g/L *w/v*) was used in all the experiments, except when otherwise indicated. The plant pathogenic oomycete *P. ultimum,* from our laboratory culture collection, was stored in sterile tubes containing Potato Dextrose Agar (PDA, Oxoid, UK) and routinely grown in Petri dishes (90 mm diameter) containing PDA at 24 °C. *Pl. viticola*, isolated from an untreated vineyard in San Michele all’Adige (Italy) in 2018, was maintained on grapevine plants (*Vitis vinifera* cv. Pinot Noir plants, grafted onto Kober 5BB) by subsequent weekly inoculations according to [18]. Briefly, grapevine plants showing oil spot symptoms were kept in the dark at 20–21 °C and 100% relative humidity (RH). After 16 h, freshly sporulating lesions on the abaxial grapevine leaf surface were gently washed with cold (4–5 °C) distilled water, and the resulting *Pl. viticola* sporangia suspension was adjusted to 2.5 × 10^5^ sporangia/mL by counting with a haemocytometer (Thoma chamber, HBG, Germany) under a light microscope. Subsequently, suspension of *Pl. viticola* sporangia was sprayed onto the abaxial surface of each fully expanded grapevine leaf using a hand sprayer. Once inoculated, plants were maintained in the greenhouse at 20 ± 0.5 °C (80–99% RH) in the dark overnight and, subsequently, moved at 25 °C (60–80% RH) with a 16/8-h day/night light regime for seven days.

### 2.2. Matrix-Assisted Laser Desorption/Ionisation-Orthogonal Time of Flight Mass Spectrometric Imaging (MALDI-qTOF-MSI)

AZ78 cells were spot-inoculated on 1 mm thick growth medium layer poured onto sterile glass slides. As an even and smooth surface is required to get reproducible conditions for MALDI-qTOF-MSI, we used our recently published microassay [27]. Briefly, two sterile glass slides were held in place at a distance of 1 mm by sterile spacers. This construct was placed in a sterile Petri dish (90 mm diameter), and 13 mL of LBA 1:10 was poured in the Petri dish, filling the gap between the slides, giving an area of 5.5 × 2.5 cm. Once solidified, the medium in excess was cut and discarded, and the glass slide on the top and the spacers were gently removed. Lastly, five μL of AZ78 cell suspension (1 × 10^8^ colony forming units [CFU]/mL) were spot-inoculated on the growth medium layer adhering to the glass slide. An area of 25 mm^2^ of growth medium was kept apart from the inoculated medium in order to identify the mass spectrometric signals (*m*/*z* values) belonging to the growth medium (blank).

Once inoculated, the glass slides were incubated at 25 °C for 36 h, and, subsequently, samples were dried in a desiccator under vacuum overnight at room temperature. Afterwards, a photograph was taken using a glass slides scanner, and 0.15–0.20 mg of a 1:1 mixture of 2,5-dihydroxybenzoic acid (2,5-DHB) and alpha-cyano-4-hydroxy-cinnamic acid (CHCA) were sublimed per cm^2^ onto the sample using home-built instrumentation. A subsequent recrystallisation step at 86 °C in an acidic environment (acetic acid solution [1% *v/v*]) for 1 min was used to ensure analyte incorporation. MALDI-qTOF-MSI experiments were immediately performed on a Synapt G2 HDMS (Waters, Milford, MA, USA) in positive linear mode, with 150 × 75 μm laser step, the laser energy was set to 250 a.u., 1000 Hz of firing rate, 1 scan per pixel and a mass range of 20–4000 Da. For accurate mass measurements, the instrument was calibrated before each run using red phosphorous. Data of three biological replicates of the microassay per treatment (growth medium) were analysed using Datacube Explorer [28], MSiReader [29] and MassLynx (Waters). The sum of the mass spectra acquired from the imaging experiment was used to search for antimicrobial-related signals. For tentative assignment of analytes to measured signals, *m*/*z* values extracted from the sum mass spectra were submitted to the “Metabolomics Workbench” database (http://www.metabolomicsworkbench.org/, 2019) [30]. 

A maximum deviation of *m*/*z* 0.05 was considered for the assignment of the analytes, while only results relevant to bacterial metabolism and *Lysobacter* sp. metabolism, in particular, were taken into consideration.

### 2.3. Evaluation of Lysobacter capsici AZ78 Cell-Free Extracts against Pythium ultimum and Rhodococcus fascians

Ten microlitres of AZ78 cell suspension (1 × 10^8^ CFU/mL) was spot-inoculated onto the centre of a Petri dish containing LBA 1:10 and incubated at 27 °C. After 48 h, Petri dishes were flooded with 3 mL of ethyl acetate, scraped with a loop, and allowed to stand for three minutes. Ethyl acetate suspensions were then collected in propylene tubes and allowed to evaporate completely under a chemical hood. Dry residues were finally suspended in 1 mL of methanol (50% *v*/*v*), filtered through 0.2 µm filters (Filtropur S 0.2, Sarstedt, Nümbrecht, DE) to obtain cell-free extracts and stored at −20 °C. Non-inoculated LBA 1:10 dishes were used as controls. Extractions were performed in triplicates (three Petri dishes), and the experiment was carried out twice.

Extracts were evaluated for their antibacterial activity against *Rhodococcus fascians* LMG 3605 and anti-oomycete activity against *P. ultimum* using a resazurin-based microtitre assay. Antibacterial assays against *R. fascians* were performed as previously described by [23]. In brief, 140 µL of LB 1:10 were added to the wells of a sterile 96-well microtitre plate and amended with 20 µL of extract. Next, 20 µL of *R. fascians* cell suspension (1 × 10^9^ CFU/mL) and 20 µL of resazurin dye solution (300 µg/mL *w*/*v*; Sigma, USA) were added to the wells. Anti-oomycete assays using *P. ultimum* were performed following the same procedure. The only difference was the replacement of *R. fascians* cell suspension volume with a plug (5 mm) of mycelium cut from the edge of two days old PDA dishes instead. 

In both assays, 96-well microtitre plates were incubated at 28 °C with continuous shaking for 7 h, and fluorescence was measured (540 nm excitation, 590 nm emission) on a microplate reader (Synergy 2 Multi-Mode Microplate Reader, BioTek, Winooski, VT, USA). The broad-spectrum antibiotic chloramphenicol and the antifungal cycloheximide were used as positive controls at the final concentrations of 25 µg/mL and 100 µg/mL (*w*/*v*), respectively. LB 1:10 seeded solely with *P. ultimum* or *R. fascians* was used as untreated controls, whereas LB 1:10 not seeded with *P. ultimum* or *R. fascians* was used as a blank. 

Relative *P. ultimum* and *R. fascians* cell viability was calculated according to the following formula:

$$Cell\;viability\;\left(\%\right)=\left(\frac{Fluorescence\;intensity\;A}{Fluorescence\;intensity\;A^{\prime }}\;\right)\times 100$$

where A is the tested extract, and A’ the untreated control. Fluorescent intensity values were normalised to the control extracts (non-inoculated plates) beforehand. Each sample was prepared in triplicate (wells), and the experiments were repeated.

### 2.4. LC-HRMS/MS Analysis of Lysobacter capsici AZ78 Cell-Free Extracts

#### 2.4.1. Sample Preparation

AZ78 culture incubated for 72 h at 27 °C was used to create a cell suspension in sterile NaCl solution 0.85% (*w*/*v*), and the absorbance was adjusted to an optical density of 0.1 absorption units at 600 nm and 1 cm path length (A_OD600_), corresponding to 1 × 10^8^ CFU/mL [20], using a spectrophotometer (UV-2450, Shimadzu, Kyoto, JP). Fifty microlitres of AZ78 cell suspension was then evenly spread on the surface of Petri dishes (90 mm diameter) with 15 mL of solid LBA 1:10 medium, using a sterile cell spreader. Inoculated LBA 1:10 with AZ78 was incubated for 72 h at 27 °C. Subsequently, media with AZ78 cultures were cut into pieces using a sterile spatula and inserted in sterile flasks with 30 mL of extraction solution consisting of MeOH, ACN and H_2_O (1.5:1.5:1) plus 0.1% formic acid (*v*/*v*). Flasks were stirred for 1 h, and the extracts were transferred to sterile 15 mL tubes. Cell-free extracts were obtained by centrifugation for 30 min at 35,000× *g*. The samples were frozen at −80 °C and lyophilised in a freeze dryer (FreeZone 6 Plus, Labconco, Kansas City, MO, USA) for 94 h. The dried samples were resolved in 3 mL of extraction solution. Five hundred microlitres from concentrated AZ78 extracts was centrifuged at 30,000× *g* for 20 min at −4 °C, and the supernatant was subsequently filtered (Millex-GV PVFD syringe filter, 0.22 μm, Merk, Darmstadt, Germany) and transferred to 2 mL HPLC vials that were stored at −80 °C until measurement.

#### 2.4.2. LC-HRMS/MS Measurement

AZ78 cell-free extracts and reference standard compounds derived from purified extracts of reference bacterial strains, namely dihydromaltophilin (HSAF), WAP-8294A2, cyclo(Pro-Val), cyclo(Phe-Pro), cyclo(Pro-Leu), cyclo(L-Pro-L-Tyr) and cyclo(D-Pro-L-Tyr), were analysed on a UHPLC system (Vanquish Duo, ThermoFisher Scientific, San Jose, CA, USA) coupled to an Orbitrap QExactive HF (ThermoFisher Scientific) equipped with an electrospray ionisation source (HESI). Following a 5 μL injection, the chromatographic separation of samples was carried out at 25 °C on a reversed-phase (RP) XBridge BEH C18 column (150 × 2.1 mm i.d., 3.5 μm particle size, Waters, Milford, MA, USA) coupled with a pre-column (C18 4 × 3 mm i.d., Security Guard Cartridge, Phenomenex, Torrance, CA, USA). The liquid phase was applied at a constant flow rate of 250 μL/min and consisted of H_2_O with 0.1% formic acid (*v*/*v*) (eluent A) and MeOH with 0.1% formic acid (*v*/*v*) (eluent B). Gradient elution was applied, starting with 90% A and 10% B held constant for 2 min, followed by a 30 min linear increase to 100% B and remaining at 100% B for 5 min, then re-equilibration at 10% B for 8 min. HESI was operated in fast polarity switching mode. The following MS and HESI parameters were used: Scan range: *m*/*z* 120–1800; sheath gas flow rate 55 arb. units, aux gas flow rate 5 arb. units with a temperature of 350 °C, spray voltage 3.5 kV (positive mode) or 3 kV (negative mode). Full MS and MS/MS measurements were carried out in separate runs using the following resolving power settings: 120,000 for FullMS mode and 60,000 for the data-dependent MS/MS mode with HCD settings: (N)CE both stepped (70 eV, 90 eV and 120 eV) and individually 100 eV and 120 eV. Reference standards and samples were measured under identical conditions within the same sequence run. The injection sequence was randomised. The stability of the instrument was checked by regular injection of QC samples during the sequence run.

#### 2.4.3. LC-HRMS/MS Data Analysis

For the identification of selected compounds, LC-HRMS data from FullMS acquisition mode were manually analysed using the open-source software MZmine [31], version-2.53 (http://mzmine.github.io/) with regard to the correspondence of accurate mass, retention time and peak shape as well as presence and intensity distribution of detectable ion species of reference standards and compounds detected in the samples. Peak shape correlation was calculated with XCMS package in R [32], v. 3.11.7, using the Pearson correlation method. MS/MS spectra interpretation included comparison and calculation of spectral similarity values of the corresponding spectra of the reference standards and the detected compounds using the OrgMassSpecR package in R, v. 0.5–3 [33]. A baseline threshold of 10% and an *m*/*z* tolerance of 0.0001 was used. The criteria used for compound identification were *m*/*z* deviation < 1 ppm, relative retention time deviation < 2.5%, peak shape similarity according to a Pearson correlation coefficient (PCC) ≥ 0.9 and MS/MS spectra similarity between the reference standard and the compound found in the samples of AZ78 extract according to a cosine phi score ≥ 95. A score value of compound identification confidence was calculated according to [34]. A minimum score value of 5.0 was assumed for confident identification of a substance on the basis of the comparison values determined with the reference standards.

### 2.5. Impact of High-Temperature Exposure on Lysobacter capsici AZ78 Cell-Free Supernatant

AZ78 cells were grown on LBA 1:10 at 27 °C for 72 h. After the incubation, Petri dishes were flooded with 5 mL of sterile distilled water and scraped using sterile spatulas. The cell suspension was transferred into sterile 15 mL tubes and centrifuged at 7000× *g* for 20 min. Cell-free supernatants were filter-sterilised using 0.2 μm filters (Filtropur S 0.2, Sarstedt, DE) and transferred into sterile 2 mL microcentrifuge tubes. Subsequently, the tubes were exposed to a thermal shock consisting of incubation at 90 °C for 20 min using a thermo mixer (T-shaker EMS100 Euro Clone, IT) followed by incubation at −20 °C for 5 min. Supernatants deriving from LBA 1:10 dishes not seeded with AZ78 were used as untreated control. 

The toxicity of AZ78 cell-free supernatants exposed to high temperature against *Pl. viticola* was tested on grapevine leaf disks. To do that, the third and fourth leaves from the top of grapevine plants grown in greenhouse conditions were collected to produce leaf discs of 18 mm diameter using a sterile cork borer. Subsequently, leaf disks were transferred onto sterilised filter paper (five foils) contained in Petri dishes (90 mm diameter) taking care to turn the abaxial leaf surface to the top. Five discs were transferred in each Petri dish, and filter papers were moistened with 7 mL of sterile distilled water. 

Once transferred, leaf disks were sprayed with heat-treated AZ78 cell-free supernatant using a hand sprayer (2 mL per Petri dish). AZ78 cell suspension (1 × 10^8^ CFU/mL), sterile distilled water and a copper-based fungicide (2 g/L Cu(OH)_2_, Coprantol Hi Bio2.0, Syngenta, CH) were used as controls. Once sprayed, Petri dishes were left for five minutes under the laminar flow cabinet to dry the droplets formed on the leaf tissues and then transferred to a growth chamber at 25 ± 1 °C.

After 24 h, leaf disks were inoculated with *Pl. viticola* sporangia by spraying a sporangia suspension (2.5 × 10^5^ sporangia/mL) using a hand sprayer (2 mL per Petri dish) and maintained in the dark at 20 ± 0.5 °C (80–99% RH). After 24 h, leaf disks were dried under a laminar flow cabinet and incubated under controlled greenhouse conditions (25 ± 1 °C; 70 ± 10% RH). After six days, the disease level was assessed using the EPPO standard scale (2004), evaluating both disease severity and disease incidence. Five Petri dishes were analysed for each treatment, and the experiment was repeated.

### 2.6. Statistical Analyses

To be statistically analysed, data from cell viability of *P. ultimum* and *R. fascians*, disease incidence and severity were log_10_ and arcsine transformed. Subsequently, the data deriving from repeated experiments were subjected to a two-way ANOVA and pooled when the experiment factor was not significant. Pooled data from cell viability of *P. ultimum* and *R. fascians* were analysed by Student’s *t*-test (α = 0.05). Data related to disease incidence and severity were analysed using one-way ANOVA, and means were compared with Tukey’s test (α = 0.01). All the statistical tests were carried out using Statistica 7.1 (StatSoft, Tulsa, OK, USA).

## 3. Results

### 3.1. Mass Spectrometric Imaging Reveals a High Diversity of Antimicrobial Analytes in Lysobacter capsici AZ78 Metabolic Profile and an Intense Metabolic Activity in the Macrocolony Outer Ring

**Figure 1:**
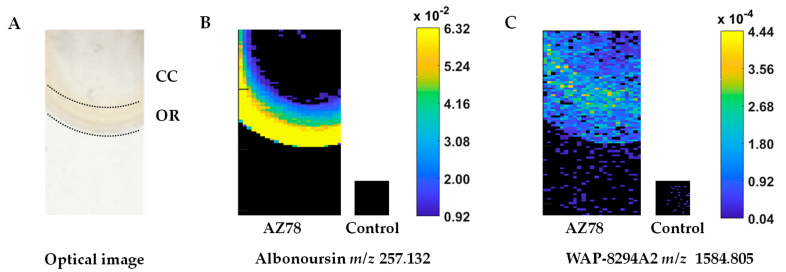
Mass spectrometric imaging of *Lysobacter capsici* AZ78 (AZ78) grown on Luria–Bertani Agar 1:10. The AZ78 macrocolony was divided into two regions of interest: the central core of the macrocolony (CC) and the outer ring of the macrocolony (OR). The optical image of the AZ78 macrocolony grown on LBA 1:10 was acquired just before matrix application (**A**); MALDI-qTOF-MS images were recorded at a lateral resolution of 150 × 75 µm and represent TIC (total ion count) normalised data corresponding to (**B**) *m*/*z* 257.132 assigned to the 2,5-diketopiperazine albonoursin and (**C**) *m*/*z* 1584.805 assigned to the cyclic lipodepsipeptide WAP-8294A2. The colour bar shows normalised intensity values (black = absent, yellow = highest intensity).

The analysis of the metabolic profile of the AZ78 macrocolony through MALDI-qTOF-MSI revealed a high diversity of signals attributed to putative antimicrobial compounds (Figure 1; Table 1). In total, nine of such signals were detected over the AZ78 macrocolony, while no respective signals were found in the media surrounding the macrocolony. Moreover, a spatial differentiation in the analyte distribution and intensity values in the AZ78 macrocolony was observed. Higher signal intensity was detected in the macrocolony outer ring (OR) compared to the central core (CC) region (Figure 1A), indicating higher metabolic activity.

A unique signal at *m*/*z* 257.132 was exclusively present in the OR region (Figure 1B). Based on accurate mass measurements, this signal was tentatively annotated as the diketopiperazine albonoursin. The remaining *m*/*z* values were shared by OR and CC regions and were tentatively assigned to different antibiotic classes (Table 1). In particular, signals at *m*/*z* 1419.866 and 1447.770 were tentatively assigned to mathemycin A and langkolide belonging to the class of macrolactone antibiotics, respectively. In addition, the latter signal (*m*/*z* 1447.770) could potentially be matched with the cyclic depsipeptide SNA-60-367-14. Furthermore, a signal at *m*/*z* 748.433 was putatively matching with the macrolide antibiotic clarithromycin. The signals at *m*/*z* 1584.805 (Figure 1C), 1600.810 and 1598.827, 1614.838 might match with the cyclic lipodepsipeptides WAP-8294A2 and WAP-8294A4 or WAP-8294Ax13, respectively. The remaining signals were putatively matching with the cyclic depsipeptides CB-182333 and CB-182348-49.

### 3.2. Lysobacter capsici AZ78 Produces Secondary Metabolites with Antioomycete and Antibacterial Activity

**Figure 2:**
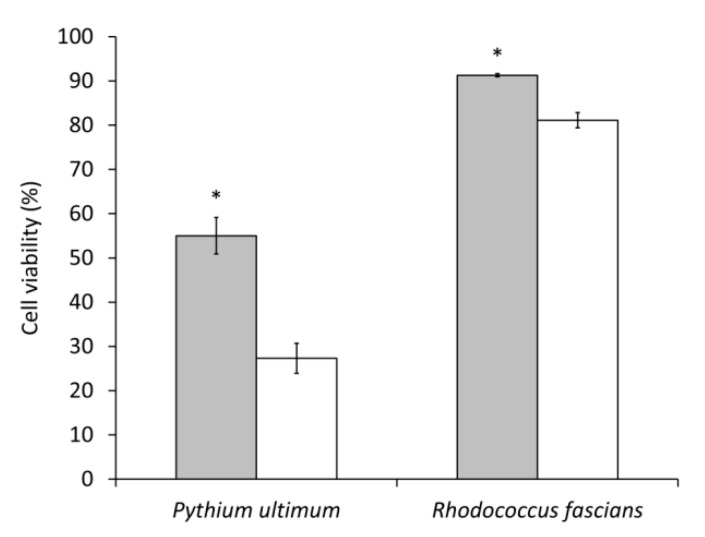
*Antimicrobial activity of Lysobacter capsici* AZ78 (AZ78) cell-free extracts. Relative percent of cell viability of *Pythium ultimum* and *Rhodococcus fascians* observed in response to AZ78 cell-free extracts (grey bars) and the references cycloheximide (100 µg/mL) for *P. ultimum* and chloramphenicol (25 µg/mL) for *R. fascians* (white bars). Mean and standard errors were calculated as the pool of three replicates from two independent experiments. Asterisks indicate significant differences according to Student’s *t*-test (α = 0.05).

The toxicity of AZ78 cell-free extracts was evaluated *in vitro* to confirm the presence of secondary metabolites active against plant pathogenic microorganisms. AZ78 cell-free extracts were able to impair the growth of *P. ultimum* and *R. fascians* (Figure 2), albeit to a lower extent than the references cycloheximide (100 µg/mL) and chloramphenicol (25 µg/mL). Upon seven hours of exposure to AZ78 cell-free extracts, *P. ultimum* viability decreased by 45% compared to the untreated control. In contrast, a low reduction in cell viability was registered in the case of *R. fascians* (9%).

### 3.3. The Cyclic Lipodepsipeptide WAP-8294A2, the Polycyclic Tetramic Acid Macrolactam Dihydromaltophilin and Four Diketopiperazines Are Confirmed as Antimicrobial Constituents of Lysobacter capsici AZ78 Metabolome

Based on the MALDI-qTOF MSI analysis and the antimicrobial activity, further investigation was performed to confirm the presence of the respective antimicrobial compounds of the initial candidate list (Table 1). In addition, the presence of other antimicrobial compounds previously reported to be produced by *Lysobacter* spp. was investigated through LC-HRMS(/MS) analysis. At first, *m*/*z* values of common ion species (e.g., [M+Na]^+^, [M+NH_4_]^+^, [M-H]^−^, [M+FA-H]^−^, etc.) assigned to the annotated compounds were searched in the HRMS spectra of AZ78 cell-free extracts from FullMS acquisition mode (Table 2). In the case of clarithromycin, mathemycin A, langkolide, SNA-60-367-14, CB-182333, CB-182349 and CB-182348, none of the predicted ion species was detected in the LC-HRMS spectra of AZ78 cell-free extracts (Table 2). In contrast, commonly known adduct ions were found for the compounds albonoursin, cyclo(Pro-Val), cyclo(Phe-Pro), cyclo(Pro-Leu), cyclo(Pro-Tyr), dihydromaltophilin, maltophilin, alteramide A, alteramide B, WAP-8294A2, WAP-8294A4 and WAP-8294Ax13. Notably, no differentiation between the adduct ions assigned to the isomers maltophilin and alteramide A, as well as WAP-8294A4 and WAP-8294Ax13, could be made based on the analysis of the HMS(/MS) spectra of AZ78 cell-free extracts.

Subsequently, AZ78 cell-free extracts and, when available, reference standards were measured by UHPLC-HRMS(/MS) under identical conditions. Chromatographic as well as spectral data of reference standards and AZ78 cell-free extracts were compared (Table 3, Figures S1A–F, S2A–F and S3). The data were screened for the presence of common ion species of the tested compounds (e.g., [M+Na]^+^, [M+NH_4_]^+^, [M-H]^−^, [M+FA-H]^−^, etc.) by inspecting the extracted ion chromatograms (EICs). In all cases, [M+H]^+^ was found to be the most abundant ion and therefore was chosen for MS/MS analysis (Table 3).

The diketopiperazines cyclo(L-Pro-L-Tyr), cyclo(Pro-Val) and cyclo(Phe-Pro) were clearly confirmed in AZ78 cell-free extracts. For these compounds, all identification criteria, including the peak shape similarity, were above the defined acceptance threshold values (Table 3). More specifically, relative mass deviations were below 1 ppm, peak shapes showed high correlation (Pearson correlation coefficient ≥ 0.96) and relative retention time deviations were less than 1% between the reference standards and AZ78 extract samples (Table 3 and Figure S1B–D). In addition, acquired LC-HRMS/MS spectra of the respective [M+H]^+^ ions showed a very high spectrum similarity (cos Φ > 0.98) (Table 3 and Figure S2B–D). Consequently, the determined compound identification score value was calculated to be 9.0, which is clearly above the threshold of 5.0 for confident identification suggested by [34].

The EIC of [M+H]^+^ for diketopiperazine cyclo(Pro-Leu) showed two closely eluting peaks in the chromatograms of AZ78 extract samples, but neither of the two perfectly co-eluted with the peak of the reference standard (Figure S1A). Nevertheless, acquired LC-HRMS/MS spectra of both substances found in the sample chromatograms showed highly similar LC-HRMS/MS spectra (cos Φ value of 1.0) to the reference standard (Figures S2A and S3). This suggests that the respective AZ78 compounds may be other stereoisomers of the reference compound cyclo(Pro-Leu). Consequently, the presence of cyclo(Pro-Leu) could be confirmed, although the precise stereochemistry of the two isomers could not be determined by the analytical method used.

The annotation of the two closely eluting compounds of AZ78 extract as stereoisomers of the diketopiperazine cyclo(Pro-Leu) (C_11_H_18_N_2_O_2_, [M+H]^+^ at *m*/*z* 211.1441) was further supported by the fragmentation pattern of their respective MS/MS spectra. More specifically, the main fragment ion found in both spectra of the sample compounds as well as the spectrum of the reference standard at *m*/*z* 70.0658 was annotated based on accurate mass as the adduct [M-C_7_H_11_NO_2_]^+^ matching the pyrrolidine ring (C_4_H_7_N), part of the prolyl residue (Figure S2A and Figure S3). Another characteristic ion fragment at *m*/*z* 72.0450 found in the MS/MS spectrum of the sample compound eluting at 12.5 min (Figure S3) was annotated as the adduct [M-C_8_H_13_NO]^+^ corresponding to one of the two amide groups (C_3_H_5_NO) of the leucyl and prolyl residue, respectively, part of the central ring of the diketopiperazine scaffold. Furthermore, the ion fragment at *m*/*z* 69.0706 found in the MS/MS spectrum of the sample compound eluting at 12.0 min (Figure S2A) may correspond to the adduct [M-C_6_H_10_N_2_O_2_]^+^ matching the isobutyl (C_5_H_8_) side chain derived from the leucyl residue. In nature, four different stereoisomers of cyclo(Pro-Leu) can be found [35]. Consequently, the slight differences observed in the MS/MS spectra of the compounds eluting at 12.0 min and 12.5 min, as far as the retention time shift from the cyclo(Pro-Leu) reference standard, can be regarded as a result of the different 3D conformations of the two isomers found in the sample and a third isomer found in the reference standard. The diketopiperazine cyclo(D-Pro-L-Tyr) was additionally analysed as a reference standard compound, but it was not found in the extracts of AZ78.

The cyclic lipodepsipeptide WAP-8294A2 and polycyclic tetramic acid macrolactam dihydromaltophilin (HSAF) were further confirmed as constituents of AZ78 metabolome, as data generated from the LC-HRMS(/MS) analysis of reference standard compounds and AZ78 extracts were consistent (Table 3 and Figure S1E,F).

### 3.4. Lysobacter capsici AZ78 Produces Dihydromaltophilin Resistant to Heat Shock and Active against Plasmopara viticola on Grapevine Leaf Disk

**Figure 3:**
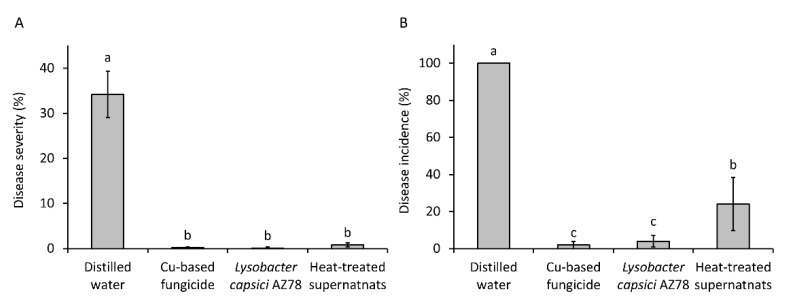
Control of *Plasmopara viticola* infection on grapevine leaf disks by the application of heat-treated *Lysobacter capsici* AZ78 (AZ78) cell-free supernatants. (**A**) Disease severity (% of leaf area covered by sporulating lesions), (**B**) Disease incidence (% of leaf disks showing symptoms). Twenty-four hours before the inoculum of *Pl. viticola*, grapevine leaf disks were treated with distilled water; copper-based fungicide; AZ78 cells; and heat-treated AZ78 cell-free supernatants. For each treatment, five Petri dishes containing five leaf disks were used. Data originating from two independent experiments were pooled and statistically analysed. Mean values ± standard deviations are reported for each treatment. Columns with the same letters do not differ significantly according to Tukey’s test (α = 0.01).

The evidence of the production of the secondary metabolite HSAF by AZ78 led us to test the effect of a heat shock on the inhibitory activity of AZ78 cell-free supernatants against *Pl. viticola*. All the grapevine leaf disks treated with distilled water showed symptoms (disease incidence = 100%), and more than 30% of the surface area (34.20 ± 5.18%) was covered with sporulating lesions (Figure 3). Both the application of the copper-based fungicide and AZ78 cells drastically reduced *Pl. viticola* attacks on grapevine leaf disks (Figure 3). Similarly, the application of AZ78 cell-free supernatants previously exposed to heat shock determined a relevant reduction in disease severity (0.80 ± 0.44%), showing no significant differences with the application of AZ78 cells and the copper-based fungicide. Differently, the application of heat-treated AZ78 cell-free supernatant was not as effective as AZ78 cells and the copper-based fungicide in reducing the percentage of grapevine leaf disks showing symptoms. However, the disease incidence observed in the case of heat-treated AZ78 cell-free supernatants (24.00 ± 14.31%) was significantly lower than the untreated control (Figure 3).

## 4. Discussion

The latest studies on AZ78 metabolic profile strongly indicated that it is abundant in various antibiotic compound classes. Several bioactive 2,5-diketopiperazines have been identified and/or isolated from extracts of AZ78 cultures grown on nutrient-rich growth media [19,23]. Furthermore, genetic and analytical results have indicated the production of other bioactive compounds by AZ78 probably related to HSAF and WAP-8294A derivatives [24]. In the present study, we further characterised the metabolic profile of the bacterium in nutrient-limiting conditions, verified the antimicrobial activity of the respective AZ78 cell extracts and identified putative antimicrobial metabolite targets.

The MALDI-TOF-MSI technique provides a rapid analysis of compounds directly desorbed from minimally processed intact cells to investigate metabolic profiles [36]. Based on this property, MALDI-TOF MSI has been used for determining the spatial distribution of certain metabolites during interactions between plant-beneficial bacteria [37], and it has been applied for the identification of plant-associated bacterial species [38,39].

In the present study, we used this technique to determine the metabolic profile of the AZ78 macrocolony developed on LBA 1/10, focusing our attention on putative antimicrobial compounds. The AZ78 macrocolony showed a phenotype characterised by the differentiation of the OR and CC regions, similar to the macrocolony phenotype observed when AZ78 was grown under rhizosphere-mimicking conditions [24]. The differentiation of the AZ78 macrocolony into two metabolically different subpopulations might be related to the scarcity of nutrients that bacterial cells face in LBA 1:10 and the rhizosphere mimicking conditions. In total, nine signals tentatively assigned to antibiotic compounds were detected. The vast majority of these analytes were found in both OR and CC of the AZ78 macrocolony with the exception of one signal tentatively matching the 2,5-diketopiperazine albonoursin that was specifically detected in the OR region. The remaining signals were assigned to a macrolide antibiotic, two macrolactones, cyclic lipodepsipeptides and cyclic depsipeptides. Based on the MALDI-qTOF-MSI analysis, organic compounds released from the AZ78 macrocolony were subsequently extracted, and antimicrobial assays were performed. The results showed that the organic phase of the cell-free extracts was effective in reducing the viability of the tested microorganisms confirming the results achieved through MALDI-qTOF-MSI analysis. In particular, AZ78 cell-free extracts were highly toxic against *P. ultimum*, whereas a lower level of toxicity was observed against *R. fascians.*

The antibacterial activity was related to the presence of the cyclic lipodepsipeptides WAP-8294A, a secondary metabolite toxic against Gram-positive bacteria The well-characterised WAP-8294A2 has a specific action against Gram-positive bacteria by targeting the menaquinone present in their cell membrane, causing membrane disruption and eventually cell lysis [40]. The low toxicity registered against *R. fascians* might be due to a low concentration of WAP-8294A in AZ78 cell extracts. Indeed, *L. enzymogenes* OH11 produce this antibacterial compound at low concentration levels and only under certain conditions [41]. The scarce production of these antibiotics by *L. enzymogenes* OH11 might represent a self-protection mechanism that this bacterial strain developed to counteract the potent toxic activity of cyclic lipodepsipeptides [42]. Thus, it is probable that the limited production of antibacterial metabolites, such as WAP-8294A, by AZ78 might be due to the absence of specific nutrient conditions and/or of Gram-positive competitors. Further analysis on cyclic lipodepsipeptides in AZ78 shall be made to confirm this hypothesis, focusing the attention on how the availability of nutrients and the presence of Gram-positive bacteria might modulate their production.

With MALDI-TOF technique, the determination of the structure of the analytes under investigation can be challenging [43]. In contrast to MALDI-TOF, LC-MS/MS technique offers more information on the structure of the metabolites. LC-MS/MS analysis of bacterial samples and synthetic compounds has been implemented in the past for the unambiguous identification of certain metabolites (i.e., N-acylhomoserine lactones) previously annotated by MALDI-TOF in bacterial samples [44]. To accurately identify the secondary metabolites released by AZ78, we used UHPLC-HRMS(/MS) to analyse AZ78 cell extracts together with reference standard compounds. In this analysis, we focused our attention on the secondary metabolites previously annotated by MALDI-qTOF and putative antimicrobial compounds reported in the literature to be produced by *Lysobacter* spp.

The signals detected by MALDI-qTOF assigned to antibiotic macrolactones, a macrolide and cyclic depsipeptides could not be confirmed by the UHPLC-HRMS analysis. This might indicate that they were probably not present in the acquired AZ78 extracts, and different sample preparation should be followed for their extraction in the future. However, the absence of these compounds supports the importance of using a MS/MS technique to confirm results achieved by using MALDI-qTOF.

After a first investigation of the LC-MS data, the presence of the diketopiperazines cyclo(L-Pro-L-Tyr), cyclo(Pro-Val), cyclo(Phe-Pro), cyclo(Pro-Leu) and albonoursin, the polycyclic tetramic acid macrolactams dihydromaltophilin (HSAF), maltophilin, alteramide A and alteramide B, as well as the presence of the cyclic lipodepsipeptides WAP-8294A2, WAP-8294A4 and WAP-8294Ax13 was supported. The four diketopiperazines, HSAF and WAP-8294A2 were further investigated since respective pure compounds could be used as reference standards. Eventually, cyclo(L-Pro-L-Tyr), cyclo(Pro-Val), cyclo(Phe-Pro), HSAF and WAP-8294A2 were confirmed in the metabolic profile of AZ78. Furthermore, two putative stereoisomers of cyclo(Pro-Leu) were further identified in the AZ78 metabolic profile. No differentiation could be made between maltophilin, a polycyclic tetramic acid macrolactam with a 5-5-6 ring system and the isomer alteramide A, with a 5-5 ring system [45,46,47]. Nevertheless, the presence of maltophilin is strongly supported, given that this compound is considered a precursor in HSAF biosynthesis [48].

Overall, UHPLC-HRMS/MS confirmed the respective MALDI-qTOF-MSI signals of the cyclic lipodepsipeptides. Indeed, the signals detected at *m*/*z* 1584.805 and *m*/*z* 1600.810 corresponded to WAP-8294A2 sodium [M+Na]^+^ and potassium [M+K]^+^ adducts, respectively. Likewise, the respective signals at *m*/*z* 1598.827 and *m*/*z* 1614.838 may be assigned more confidently to WAP-8294A4 or WAP-8294Ax13 [M+Na]^+^ and [M+K]^+^ adducts since the production of its chemical analogue WAP-8294A2 was confirmed. It is worth mentioning that although WAP-8294A4 and WAP-8294Ax13 constitute homologues of WAP-8294A2, differing by one CH_2_- group [49], the respective data acquired by the HRMS/MS analysis could not support the differentiation between these isomers. 

The production of WAP-8294A2 by AZ78 places *L. capsici* as an additional species in *Lysobacter* genus producing this cyclic lipodepsipeptide compound family. WAP-8294A2 showed remarkable inhibitory activity against *Bacillus* sp. and *Staphylococcus* sp. (MIC of 2 μg/mL *w*/*v*) [40]. Notably, this antibiotic compound is active against drug-resistant clinical *S. aureus* isolates and is currently being developed as a pharmaceutical drug against systemic MRSA (methicillin-resistant *S. aureus*) infections [50]. Thus, members of *L. capsici* species might be taken into consideration in the future for the production of cyclic lipodepsipeptides to be developed as pharmaceutical drugs.

Previously, an analyte putatively associated with WAP-8294A2 was identified in the metabolic profile of the AZ78 macrocolony grown on a nutrient-rich growth medium (LBA), while no WAP-8294A2 was found when a growth medium mimicking the rhizosphere composition was used [24]. Even if LBA 1:10 constitutes a growth medium limited in nutrients, its composition is profoundly different from the rhizosphere-mimicking medium used by [24]. Indeed, LBA 1:10 contains tryptone (1 g/L *w*/*v*) that is a protein source more abundant than the protein content of the rhizosphere-mimicking medium. Recently, it was shown that protein-rich growth media can enhance antibiotic production in *Streptomyces* sp. SD1 [51]. Consequently, it is possible that the availability of proteins in LBA 1:10 activated the production of the metabolically expensive secondary metabolite WAP-8294A2 by AZ78.

The polycyclic tetramic acid macrolactams have shown specific suppressive activity against plant pathogenic fungi and oomycetes. Alteramide B, isolated from *L. enzymogenes,* was shown to inhibit various plant pathogenic fungi and oomycetes in vitro [52], while the application of dihydromaltophilin (HSAF) successfully controlled *Fusarium* head blight in wheat [53] and post-harvest application of HSAF in pears reduced anthracnose symptoms caused by *Colletotrichum fructicola* [54]. Since heat-treated AZ78 extracts showed prophylactic activity against *Pl. viticola* when applied on grapevine leaf disks, we may confidently confirm the presence of HSAF in the AZ78 metabolome. Recent results showed that *L. enzymogenes* OH11 releases HSAF through the production of outer membrane vesicles that may deliver this antifungal compound closer to the cell walls of plant pathogenic fungi [55]. Thus, it would be interesting to determine in the future if the mechanism of HSAF delivery is a trait conserved in *Lysobacter* spp.

HSAF is a robust antifungal agent that has been shown to restrict the growth of fungi by inducing cell wall thickening due to the disruption of the biosynthesis of sphingolipids [56] and additionally by causing cell apoptosis through the induction of reactive oxygen species production [57]. Hence, the production of HSAF by AZ78 can be regarded as a specific mechanism for fungal growth inhibition. On the other hand, it has been speculated that since HSAF induces the synthesis of fungal cell wall constituents, its production will increase the carbon pool that will be available after predation and fungal cell wall lysis that constitute the common feeding strategies of bacteria, such as AZ78 [56]. Consequently, the production of HSAF by AZ78 might serve more than one purpose.

The identification of HSAF in AZ78 extracts from LBA 1:10 cultures is in accordance with former studies. HSAF has been reported to be exclusively produced by *L. enzymogenes* C3 on nutritionally limited growth media, such as TSB (tryptic soy broth) 1:10 [17]. Similarly, signals putatively assigned to HSAF and maltophilin in the metabolic profile of AZ78 were detected specifically in AZ78 macrocolonies grown on a rhizosphere-mimicking agar with restricted nutrient content [24].

In contrast, the 2,5-diketopiperazines cyclo(L-Pro-L-Tyr), cyclo(L-Pro-L-Val), cyclo(L-Pro-L-Phe) and cyclo(L-Pro-L-Leu) were previously identified as toxic secondary metabolites released by AZ78 cells on growth media with rich nutrient content. Particularly, cyclo(L-Pro-L-Tyr) was toxic against sporangia of *Ph. infestans* and *Pl. viticola* [19], while cyclo(L-Pro-L-Val) and cyclo(L-Pro-L-Phe) were toxic against the plant pathogenic bacterium *R. fascians* [23]. Additionally, cyclo(L-Pro-L-Leu) has been found with toxic activity against several fungi, including the plant pathogen *Rhizoctonia solani* [58]. Notably, albonoursin, annotated in AZ78 extracts, is a dehydro-derivative of cyclo(L-Phe–L-Leu) produced by *Streptomyces* sp. that showed antibacterial and antitumor activity [59,60].

Our present results indicate that AZ78 cells secrete the 2,5-diketopiperazines cyclo(L-Pro-L-Tyr), cyclo(L-Pro-L-Val), cyclo(L-Pro-L-Phe) and cyclo(L-Pro-L-Leu) additionally in nutritionally limited growth media. Considering that these metabolites have shown a broad-spectrum antimicrobial activity [19,23,58,61] and that their production seems not to be dependent on nutrient availability, it is conceivable that probably they are also involved in other biological mechanisms. Indeed, it has been reported that diketopiperazines might play a role in the bacterial interspecies communication as in the case of cyclo(L-Pro-L-Tyr) and cyclo(L-Pro-L-Leu) that might interact with quorum sensing systems based on N-acyl homoserine lactones in Gram-negative bacteria [32,62,63]. Thus, it would be interesting to investigate the role played by diketopiperazines in the interaction of AZ78 with other Gram-negative bacteria in the future.

## 5. Conclusions

In conclusion, our results confirmed that the AZ78 arsenal is equipped with diverse antimicrobial metabolites, including the cyclic lipodepsipeptide WAP-8294A2, the polycyclic tetramic acid macrolactam dihydromaltophilin (HSAF) and four 2,5-diketopiperazines. Although the presence of other antibiotic compounds, such as albonoursin, alteramide B and WAP-8294A4, was supported by our findings, further investigations shall be made in the future to confirm their identity since reference compounds were not available for their identification at the moment. Overall, our findings contributed to finely determine the classes of antimicrobial secondary metabolites involved in AZ78 inhibitory activity. Notably, all the identified secondary metabolites have not been reported to be toxic to humans, animals and plants. This information might be used for the further development and registration of AZ78 as a microbial biopesticide in the future. 

## Figures and Tables

**Table 1:** Initial list of antimicrobial candidate analytes created following Matrix-Assisted Laser Desorption/Ionisation-Orthogonal Time of Flight Mass Spectrometric Imaging (MALDI-qTOF-MSI) of the *Lysobacter capsici* AZ78 (AZ78) macrocolony.

Measured ^a^	Annotated			
*m*/*z*	Adduct Ion ^b^	Molecular Formula	Compound Name	Compound Class
257.132	[M+H]^+^	C_15_H_17_N_2_O_2_	Albonoursin	2,5-diketopiperazine
748.433	[M+H]^+^	C_38_H_70_NO_13_	Clarithromycin	Macrolide
1419.866	[M+Na]^+^	C_71_H_132_N_2_O_24_Na	Mathemycin A	Macrolactone
1447.770	[M+H]^+^	C_75_H_115_O_27_	Langkolide	-
	[M+H]^+^	C_72_H_111_N_12_O_19_	SNA-60-367-14	Cyclic depsipeptide
1584.805	[M+Na]^+^	C_73_H_111_N_17_O_21_Na	WAP-8294A2	Cyclic lipodepsipeptide
1598.827	[M+Na]^+^	C_74_H_113_N_17_O_21_Na	WAP-8294A4/WAP-294Ax13 *	-
1600.81	[M+K]^+^	C_73_H_111_N_17_O_21_K	WAP-8294A2	-
1614.838	[M+K]^+^	C_74_H_113_N_17_O_21_K	WAP-8294A4/WAP-8294Ax13 *	-
1650.784	[M+Na]^+^	C_72_H_109_N_17_O_26_Na	CB-182333	Cyclic depsipeptide
	[M+Na]^+^	C_72_H_109_N_17_O_26_Na	CB-182349	-
	[M+K]^+^	C_72_H_109_N_17_O_25_K	CB-182348	-

^a^ The macrocolony of AZ78 on Luria-Bertani Agar 1:10 was analysed by a MALDI-qTOF-MSI using a Synapt G2 HDMS system; ^b^ a maximum deviation of *m*/*z* ± 0.05 was considered for assigning the adduct ions to putative analytes using the Metabolomics Workbench database (https://www.metabolomicsworkbench.org/, 2019); * isomers.

**Table 2:** List of the antimicrobial compounds and respective ion species found in the high-resolution mass spectra of *Lysobacter capsici* AZ78 (AZ78) extracts following ultra-high pressure liquid chromatography–high-resolution mass spectrometry (UHPLC-HRMS) analysis.

Compound Class	Compound Name	Detected Ion Species ^3^
2,5-diketopiperazine	Albonoursin ^1^	[M+H]^+^, [M-H_2_O+H]^+^
-	Cyclo(Pro-Val) ^2^	[M+H]^+^, [M+Na]^+^
-	Cyclo(Phe-Pro) ^2^	[M+H]^+^, [M+Na]^+^
-	Cyclo(Pro-Leu) ^2^	[M+H]^+^, [M+Na]^+^
-	Cyclo(Pro-Tyr) ^2^	[M+H]^+^, [M+Na]^+^
Polycyclic tetramic acid	Dihydromaltophilin ^2^	[M+H]^+^, [M-H]^−^, [M+Na]^+−^
Macrolactam	Maltophilin/Alteramide A ^2^ *	[M+H]^+^, [M-H]^−^, [M+K]^+^, [M+Na]^+^
-	Alteramide B ^2^	[M+H]^+^, [M-H]^−^, [M+Na]^+^, [M+NH_4_]^+^
Cyclic lipodepsipeptide	WAP-8294A2 ^1^	[M+K]^+^, [M+Na]^+^, [M+H]^+^, [M-H]^−^
-	WAP-8294A4/WAP-8294Ax13 ^1^ *	[M+K]^+^, [M+Na]^+^, [M+H]^+^, [M-H]^−^

AZ78 extracts from Luria–Bertani Agar 1:10 medium were analysed on a UHPLC coupled to an Orbitrap QExactive HF equipped with a ESI source; ^1^ compounds previously annotated by MALDI-qTOF-MS (Table 1); ^2^ compounds reported in the literature to be produced by *Lysobacter* spp.; ^3^ adduct ions found in the UHPLC-HRMS spectra of AZ78 extracts in FullMS scan mode, a *m*/*z* deviation of 1 ppm was considered; * isomers.

**Table 3:** Results from the in parallel ultra-high performance liquid chromatography-high resolution tandem mass spectrometry (UHPLC-HRMS/MS) analysis of *Lysobacter capsici* AZ78 (AZ78) extracts and reference standards.

Compound	Molecular Formula	*m*/*z* Deviation [ppm] ^a^	RT Deviation [%] ^b^	Peak Shape Similarity [PCC] ^c^	MS/MS Similarity [cos Φ] ^d^	Compound Identification Score ^e^
Dihydromaltophilin	C_29_H_40_N_2_O_6_	0.61	0.09	0.91	0.97	Z|
WAP-8294A2	C_73_H_111_N_17_O_21_	0.87	0.44	0.91 ^f^	0.97	9.0
Cyclo(Pro-Val)	C_10_H_16_N_2_O_2_	0.36	0.16	0.99	0.99	9.0
Cyclo(Phe-Pro)	C_14_H_16_N_2_O_2_	0.77	0.23	0.98	1.00	9.0
Cyclo(Pro-Leu) putative isomer at RT 12.0 min	C_11_H_18_N_2_O_2_	0.71	1.63	0.85 ^f^	1.00	8.0
Cyclo(Pro-Leu) putative isomer at RT 12.5 min	C_11_H_18_N_2_O_2_	0.62	2.13	0.46 ^f^	1.00	8.0
Cyclo(L-Pro-L-Tyr)	C_14_H_16_N_2_O_3_	0.88	0.59	0.96	0.99	8.0
Cyclo(D-Pro-L-Tyr) *	C_14_H_16_N_2_O_3_	_	_	_	_	_

* This compound was not detected in AZ78 extract; ^a^ High-resolution *m*/*z* deviation between reference standard and sample; ^b^ Relative retention time deviation between reference standard and sample; ^c^ Chromatographic peak shape similarity between reference standard and sample according to Pearson correlation coefficient; ^d^ MS/MS spectrum similarity between reference standard and sample according to cosine phi; ^e^ Score for confident identification calculated according to [34]. ^f^ After retention time alignment of sample peak to reference standard peak.

## Supplementary Materials

The following are available online at https://www.mdpi.com/article/10.3390/microorganisms9061320/s1, Figure S1: Overlay of the extracted ion chromatograms of reference standards (blue) and compounds detected in AZ78 extracts (red); (A) cyclo(Pro-Leu), (B) cyclo(L-Pro-L-Tyr), (C) cyclo(Phe-Pro), (D) cyclo(Pro-Val), (E) WAP-8294A2 and (F) dihydromaltophilin; * peaks selected for further MS/MS analysis., Figure S2: Side by side comparison of LC-HRMS/MS spectra of reference standards (blue) and compounds detected in AZ78 extracts (red); (A) cyclo(Pro-Leu) at 12.0 min, (B) cyclo(L-Pro-L-Tyr), (C) cyclo(Phe-Pro), (D) cyclo(Pro-Val), (E) WAP-8294A2 and (F) dihydromaltophilin., Figure S3: Side by side comparison of LC-HRMS/MS spectra of cyclo(Pro-Leu) peak at 12.5 min (red) and reference standard (blue).
