# Supplementary material for: Characterisation of the Antibiotic Profile of Lysobacter capsici AZ78, an Effective Biological Control Agent of Plant Pathogenic Microorganisms
Source: Microorganisms. 2021 Jun 17;9(6):1320. doi: 10.3390/microorganisms9061320 (PMC8235233; doi:10.3390/microorganisms9061320)
Supplement: Supplementary file 1 [file microorganisms-09-01320-s001.zip › microorganisms-1242317-supplementary.pdf]

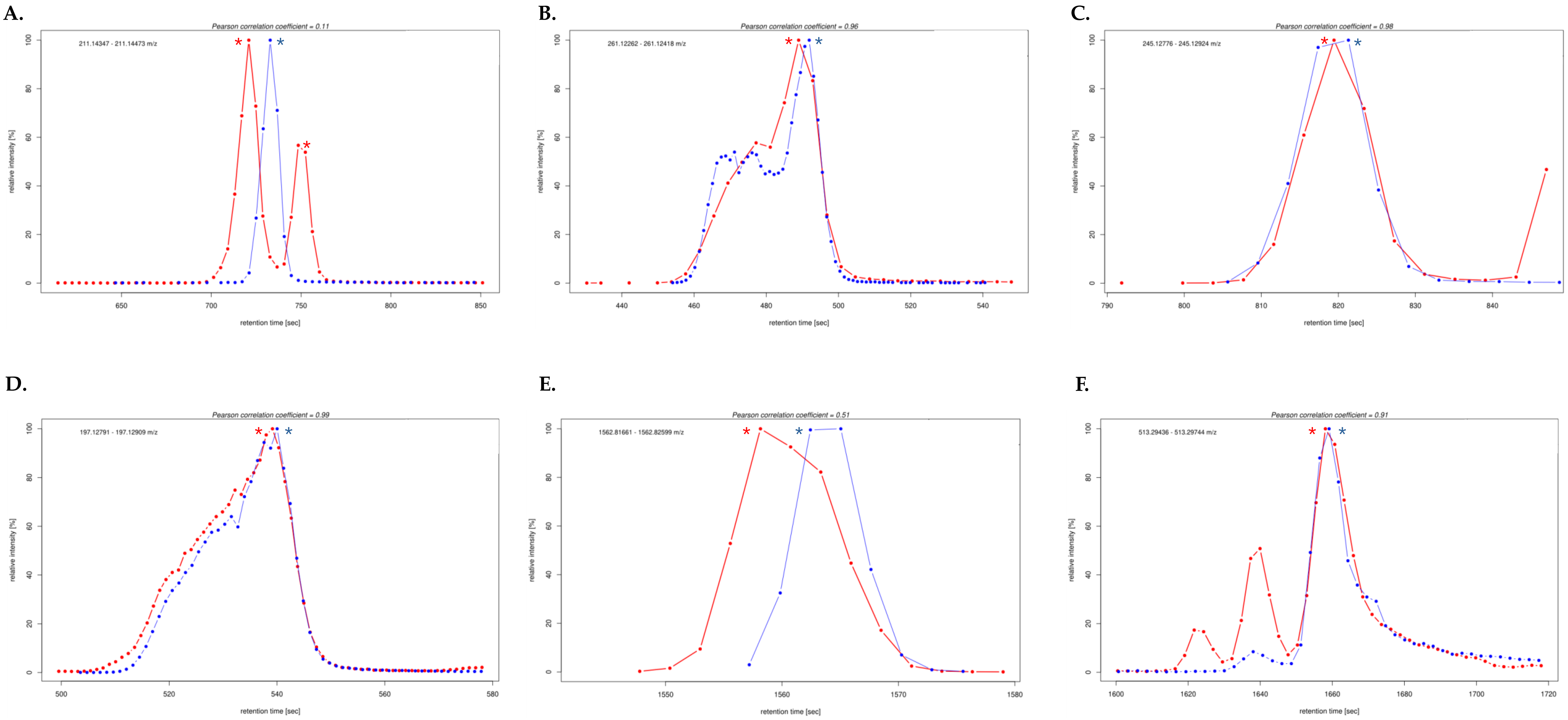

Figure S1 Overlay of the extracted ion chromatograms of reference standards (blue) and compounds detected in AZ78 extracts (red); (A) cyclo(Pro-Leu), (B) cyclo(L-Pro-L-Tyr), (C) cyclo(Phe-Pro), (D) cyclo(Pro-Val), (E) WAP-8294A2 and (F) dihydromaltophilin; * peaks selected for further MS/MS analysis.

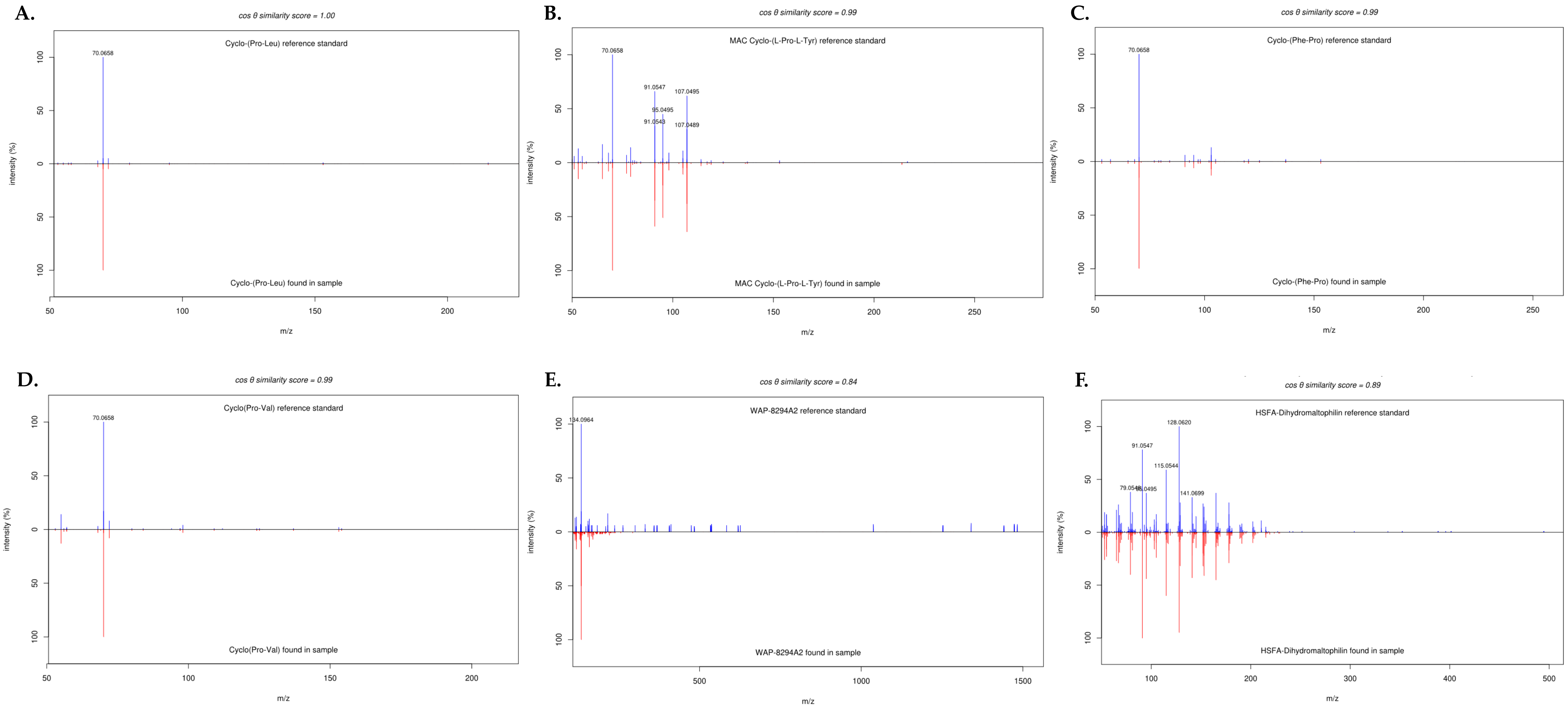

Figure S2 Side by side comparison of LC-HRMS/MS spectra of reference standards (blue) and compounds detected in AZ78 extracts (red); (A) cyclo(Pro-Leu) at 12.0 min, (B) cyclo(L-Pro-L-Tyr), (C) cyclo(Phe-Pro), (D) cyclo(Pro-Val), (E) WAP-8294A2 and (F) dihydromaltophilin.

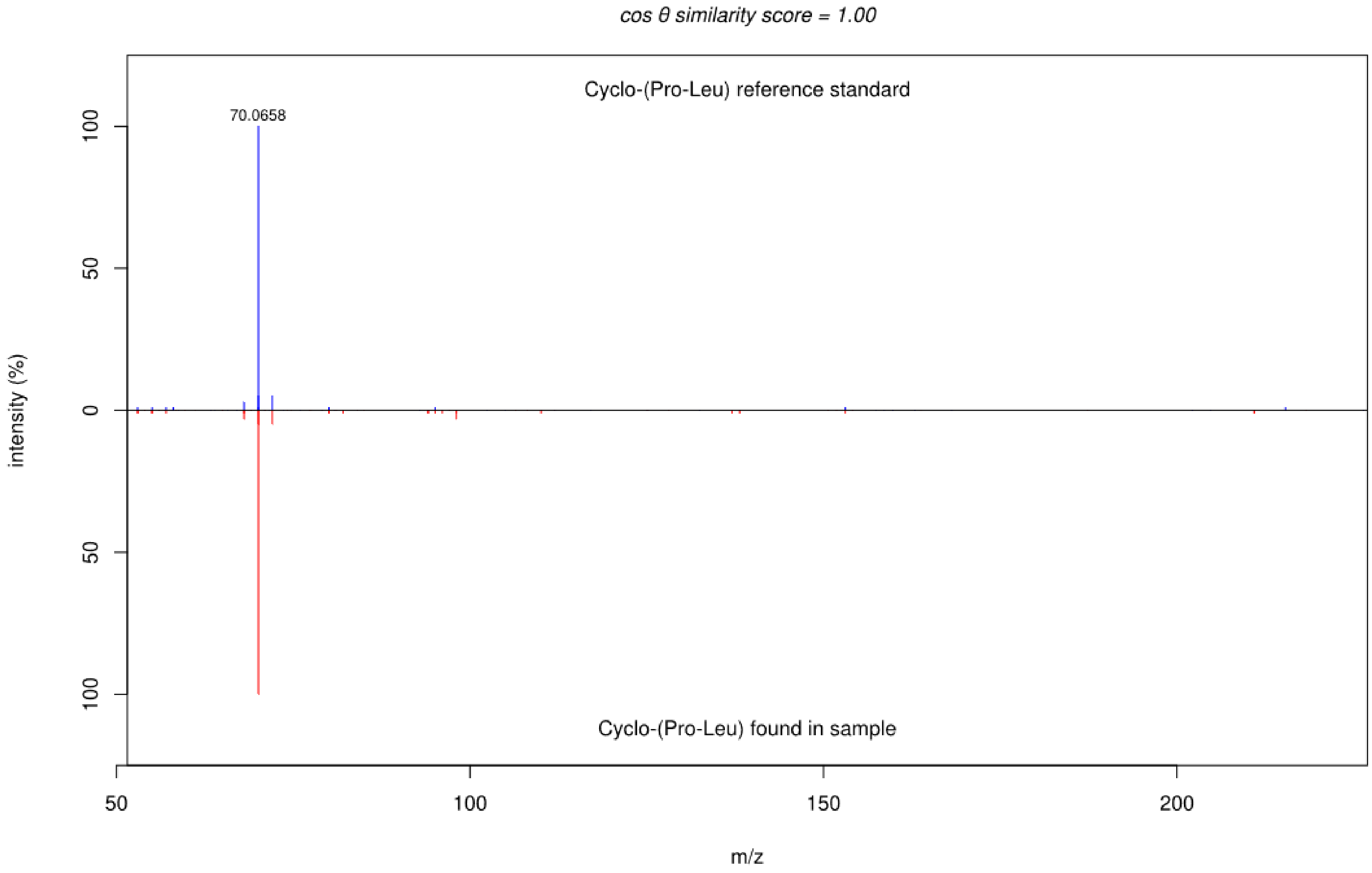

Figure S3 Side by side comparison of LC-HRMS/MS spectra of cyclo(Pro-Leu) peak at 12.5 min (red) and reference standard (blue).
